# Systemic oncolytic adenovirus delivered in mesenchymal carrier cells modulate tumor infiltrating immune cells and tumor microenvironment in mice with neuroblastoma

**DOI:** 10.18632/oncotarget.27401

**Published:** 2020-01-28

**Authors:** Lidia Franco-Luzón, África González-Murillo, Cristina Alcántara-Sánchez, Lorena García-García, Maryam Tabasi, Ana Luis Huertas, Louis Chesler, Manuel Ramírez

**Affiliations:** ^1^Fundación Oncohematología Infantil, Madrid, Spain; ^2^Unidad de Terapias Avanzadas, Oncología, Hospital Infantil Universitario Niño Jesús, Madrid, Spain; ^3^Fundación de Investigación Biomédica, Hospital Infantil Universitario Niño Jesús, Madrid, Spain; ^4^Servicio de Cirugía, Hospital Infantil Universitario Niño Jesús, Madrid, Spain; ^5^Instituto de Investigación Sanitaria La Princesa, Madrid, Spain; ^6^Paediatric Solid Tumour Biology and Therapeutics Team, Division of Clinical Studies and Cancer Therapeutics Division, The Institute of Cancer Research, Sutton, Surrey, UK

**Keywords:** oncolytic virotherapy, neuroblastoma, mesenchymal stem cells, tumor microenvironment, immune response

## Abstract

Celyvir (autologous mesenchymal cells -MSCs- that carry an oncolytic adenovirus) is a new therapeutic strategy for metastatic tumors developed by our research group over the last decade. There are limitations for studying the immune effects of human oncolytic adenoviruses in murine models since these viruses do not replicate naturally in these animals. The use of xenografts in immunodeficient mice prevent assessing important clinical aspects of this therapy such as the antiadenoviral immune response or the possible intratumoral immune changes, both of tumor infiltrating leukocytes and of the microenvironment. In our strategy, the presence of MSCs in the medicinal product adds an extra level of complexity. We present here a murine model that overcomes many of these limitations. We found that carrier cells outcompeted intravenous administration of naked particles in delivering the oncolytic virus into the tumor masses. The protection that MSCs could provide to the oncolytic adenovirus did not preclude the development of an antiadenoviral immune response. However, the presence of circulating antiadenoviral antibodies did not prevent changes detected at the tumor masses: increased infiltration and changes in the quality of immune cells per unit of tumor volume, and a less protumoral and more inflammatory profile of the tumor microenvironment. We believe that the model described here will enable the study of crucial events related to the immune responses affecting both the medicinal product and the tumor.

## INTRODUCTION

Celyvir (autologous mesenchymal cells -MSCs- that carry an oncolytic adenovirus [1] inside) is a new therapeutic strategy for metastatic tumors developed by our research group over the last decade. The first clinical trial with Celyvir [2] for children and adults with metastatic and refractory solid tumors (ClinicalTrials Identifier: NCT01844661; EudraCT2008-000364-16) and a compassionate use program [3] have shown that Celyvir is a very well tolerated treatment, with only mild toxicities related to the adenoviral infusion (fever, chills and discomfort) with the potential to achieve clinical responses in patients with advanced tumors.

Nowadays it is assumed that oncolytic virotherapy can be considered as a form of cancer immunotherapy [4], since the reported clinical benefits have been associated with antitumor immune phenomena initiated by infection and oncolysis. Results of preclinical models and human trials demonstrate that the localized effect of oncolytic viruses is capable of activating an inflammatory immune infiltrate in tumors. This point is of vital importance, since it is known that tumor infiltration by T lymphocytes is a prerequisite for the success of immunotherapies based on inhibitors of immune checkpoints [5]. Oncolytic virotherapy, and therefore Celyvir, appears as a strategy capable of achieving tumor infiltration by lymphocytes in any type of tumor, in principle. In addition to its action on tumor infiltrating leukocytes, oncolytic virotherapies can also act on the tolerant state of the tumor microenvironment [6].

There are limitations for studying the immune effects of human oncolytic adenoviruses in animal models since these viruses do not replicate naturally in murine models. The use of xenografts in immunodeficient mice allows the analysis of aspects related to oncolysis or tumor targeting but no other important clinical facets such as the antiadenoviral immune response or the possible intratumoral immune changes, both of tumor infiltrating leukocytes and of the microenvironment. In addition to the importance of the various immune reactions associated with oncolytic virotherapy, our strategy incorporates the well-known immunomodulatory role of mesenchymal stem cells (MSCs) [7–14]. We have explored the possibility of an immunocompetent murine model of neuroblastoma, a childhood tumor treated with Celyvir by our group. In this model, we comprehensively analyzed biodistribution of Celyvir, adenoviral levels in peripheral blood, antiadenoviral response and intratumor immune changes associated with repeated administrations of an oncolytic adenovirus carried by MSCs. We found that carrier cells outcompeted intravenously (IV) administration of naked particles in delivering the oncolytic virus into the tumor masses. The protection that MSCs could provide to the oncolytic adenovirus did not preclude the development of an antiadenoviral immune response. However, the presence of circulating antiadenoviral antibodies did not prevent the changes detected at the tumor masses. Therapy caused an increased infiltration and changes in the quality of immune cells per unit of tumor volume when compared to untreated mice. Tumor microenvironment showed a less protumoral and more inflammatory profile after treatment. We believe that the model described here will help us in optimizing this type of therapy by enabling the study of crucial events related to the immune responses affecting both the medicinal product and the tumor.

## RESULTS

### Mutant oncolytic adenovirus dlE102 replicates efficiently in adipose-derived murine MSC

To generate mCelyvir (murine version for human Celyvir used in the clinical setting [1, 3]), mesenchymal stem cell cultures were obtained from the white adipose tissue of mice, and were characterized by flow cytometry as described in Math & Methods. The oncolytic murine adenovirus MAV-1 dlE102 was previously developed by Dr. Katherine Spindler’s group [15]. Murine MSCs (mMSCs) were infected with mAd and viral replication was assessed by quantitative PCR (qRT-PCR). Detection of viral particles in the supernatants collected at days 2 and 7 after infection increased over time (Supplementary Figure 1A, 1B, 1C). The cytopathic effect was followed by daily visual inspection. These findings are similar to those in the human system and allowed us to study the immune effects of murine Celyvir in an immunocompetent model of neuroblastoma.

### Changes on adenovirus immunity upon systemic administration of murine Celyvir correlated with the presence of mAd particles in PB

**Figure 1:**
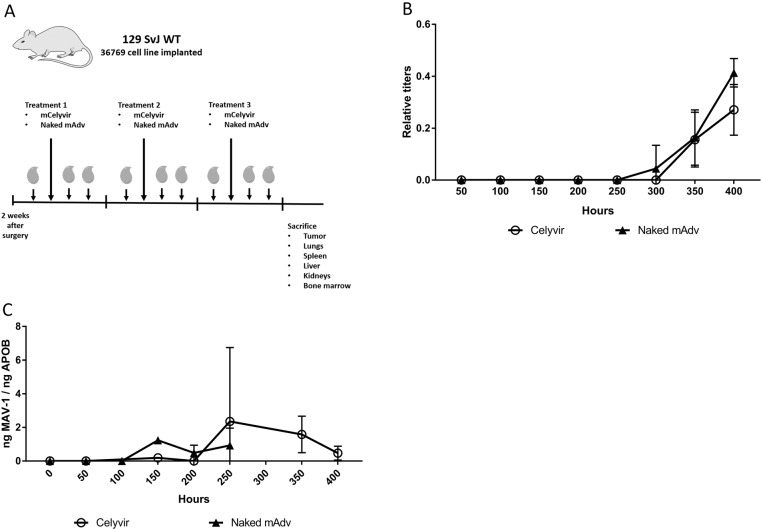
Kinetic of circulating anti-adenovirus antibodies and viral particles following repeated infusions of mCelyvir. **(A)** Schematic representation of the experimental procedure. **(B)** MAV-1 relative quantitation of serum anti-adenovirus antibody levels of mice treated with mCelyvir or naked mAd. **(C)** qRT-PCR amplification of MAV-1 specific sequence from peripheral blood samples of the same mice.

Human Celyvir is administered weekly to patients so medicine biodistribution, virus replication and antivirus immune responses are important aspects of this therapy. Tumor bearing mice were treated during 3 weeks with mCelyvir (n=5) or naked virus (n=6) and PB samples were recovered before and one / two days after each treatment aiming at assessing the systemic immune response against the oncolytic virus. A scheme of experimental procedure is represented in Figure 1A. The anti-MAV neutralizing antibodies kinetic in serum showed that all treated mice had a positive titer of specific anti-adenovirus antibody while none of the untreated one did (Figure 1B). Interestingly, antibody levels were not detected until the third dose of either naked mAd or mCelyvir, being higher for the former compared with mCelyvir. Therefore, a systemic humoral immune response against the oncolytic virus followed the repeated administration of mCelyvir.

We also analyzed the presence of mAd virus in PB samples from mice treated with either naked virus or mCelyvir. Samples from the first week were all negative. mAd genomic sequences were detectable in samples from the second week of treatment. We observed that particles of mAd decreased in both groups from the third week on, being only detectable in mCelyvir condition. Thus, circulating viral particles dropped when the titers of neutralizing anti-MAV1 antibodies raised (Figure 1C).

### Anatomical distribution of systemically administered oncolytic adenovirus delivered in carrier mesenchymal stem cells

**Figure 2:**
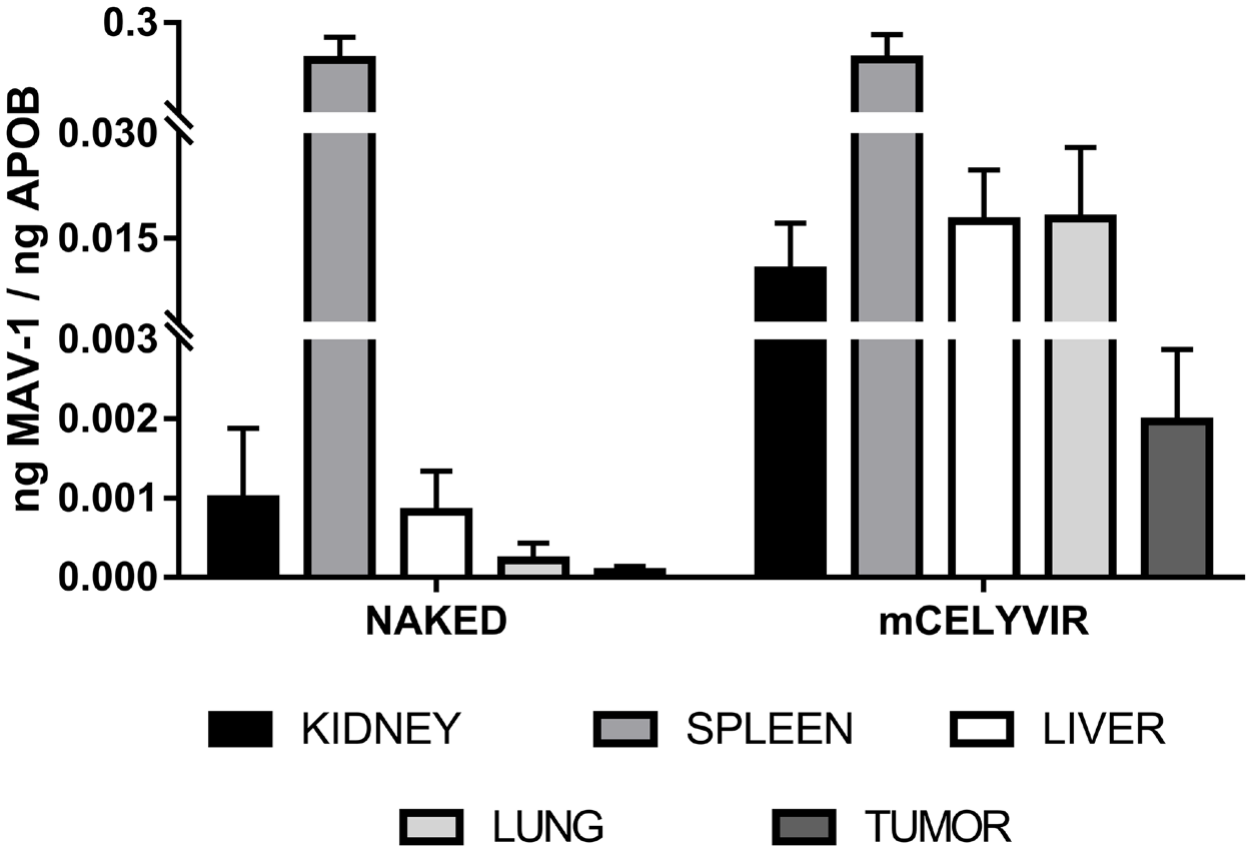
Differential biodistribution of MAV-1 in organs. qRT-PCR amplification of MAV-1 specific sequence from several organs and tumor recovered from mice treated with either naked MAV-1 or mCelyvir.

We assessed the biodistribution of the oncolytic adenovirus after repeated intravenous infusions. The mice of the experiment explained previously were exsanguinated and sacrificed one week after the fourth intravenous dose of either naked mAd or mCelyvir. Several organs and the tumor masses were procured. The administration of the oncolytic adenovirus in carrier cells resulted in higher accumulation of the virus in the tumor masses (p=0.08) and lungs (p=0.09), compared to the infusions of naked viruses. Although differences are not statically significant, there exists a tendency towards increased viral particles accumulation when using mCelyvir compared to naked viruses. No differences were found in spleens, livers and kidneys. The spleens and the lungs were the organs in which the highest amount of virus was detected (Figure 2).

### Changes on local tumor immunity upon systemic administration of murine Celyvir in a spontaneous model of neuroblastoma

**Figure 3:**
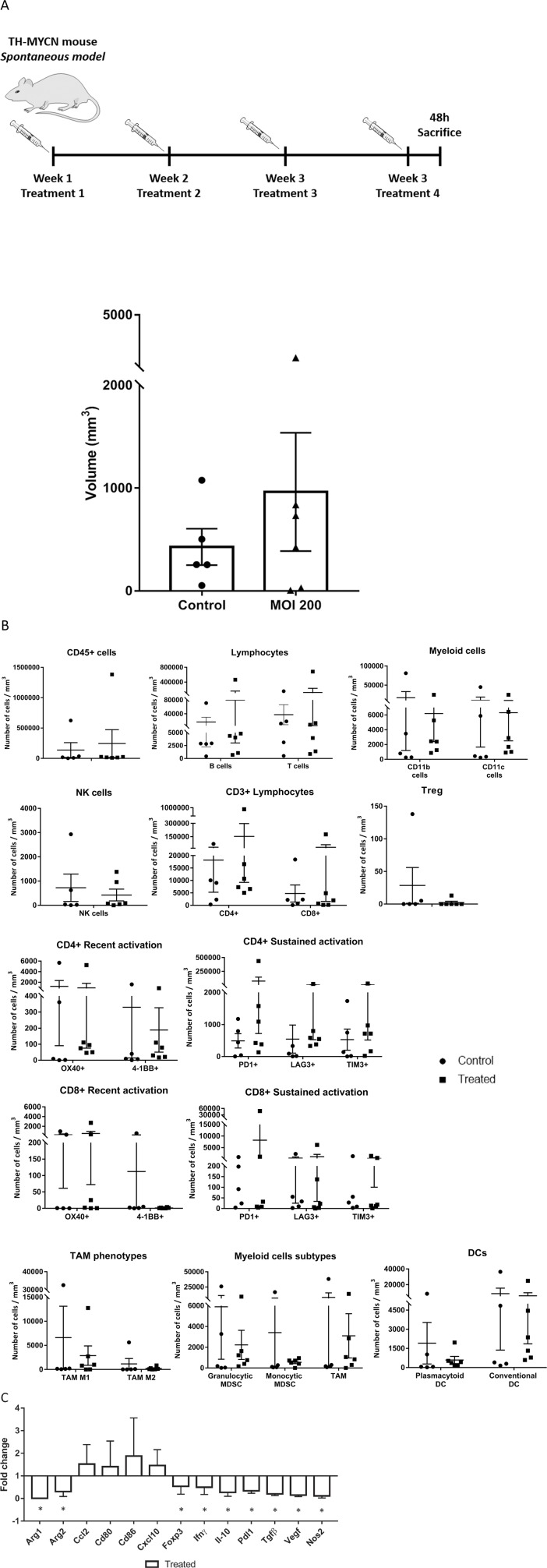
Effects of systemically administered mCelyvir in tumors in a spontaneous model of NB. **(A)** Tumor volumes recovered after four weekly treatments of intravenous mCelyvir and untreated controls. **(B)** Infiltrating immune cells per milliliter of tumor in both groups of animals. **(C)** Expression levels of immune-related genes of tumor microenvironment analyzed by qRT-PCR. Results were normalized to untreated controls (normalized value = 1). Statistics differences are represented by ^*^ (p < 0.05) or ^**^ (p < 0.01).

We next studied the local effects of IV administration of mCelyvir in tumor growth, intratumoral immune cell infiltration and expression of tumor microenvironment molecules, using the TH-MYCN transgenic mouse model (spontaneous model) as explained in Materials and Methods. The frequency of tumors was not significantly different among treated and untreated groups (5 out of 19 vs. 6 out of 15, untreated vs. treated, p>0.1), similar to what it has been reported for this model [16]. Considering only the animals with macroscopic tumors, we found high variability on tumor volumes (Figure 3A, Supplementary Figure 2). Tumors were recovered and carefully minced. Cells were counted and fresh cell suspensions were labeled with specific flow cytometry antibodies in order to analyze tumor infiltrating immune cell populations. Complete flow cytometry strategy is summarized in Supplementary Figure 3. We normalized the number of immune cells per milliliter of tumor to allow for comparisons. Even though no significant differences were found, tumors from treated mice showed a trend towards higher immune infiltration compared to untreated ones (Figure 3B). This difference was related to a higher infiltration of lymphocytes of the adaptive immune system, T- and B-lymphocytes (CD45+ CD3+ and CD45+ B220+, respectively), while myeloid (CD45+, CD11b+) and dendritic cell (CD45+, CD11c+) infiltration tended to be higher among untreated mice. All subsets of myeloid (MDSC-granulocytic, MDSC-monocytic, M1-TAM, M2-TAM) and DC (plasmacytoid and conventional) showed higher numbers per mL in the control group. On the other hand, systemic therapy with mCelyvir resulted in non-significant increased mean values of CD8 and CD4 T lymphocytes per tumor volume, expressing markers of recent and sustained T cell activation (OX40 among CD8, PD1 and LAG3 among both CD4 and CD8, and TIM3 among CD4). CD4 and CD8 T lymphocytes expressing PD1 were enriched 150 and 33 times respectively, relative to untreated mice (Supplementary Table 2). At the same time, we did not find differences in the percentages of these leukocyte subsets in peripheral blood. (Supplementary Figure 4).

We also quantified the expression levels of several genes related with immune function as mean of assessing the TME status and changes related with therapy. We found that treated mice expressed significant lower levels of genes associated to a protumoral TME (*Arg1, Arg2, Foxp3, Il-10, Tgfβ, Vegf, Nos2*) and higher (but non-significantly) levels of genes associated to a sustained lymphocyte activation (*CD80* and *CD86*, both ligands of *CTLA-4*) and a more inflammatory environment (chemokines *CCL2* and *CXCL10*) (Figure 3C).

### Changes on local tumor immunity upon systemic administration of murine Celyvir in an induced model of neuroblastoma

The fact that a significant number of spontaneous TH-MYCN animals did not develop tumor during the experimental period, and among those that did, the time of tumor initiation may not be homogeneous, contributed to the high variability found in the previous experiments. Therefore, the impact of therapy may not be comparable among groups. As alternative, we used an induced tumor model similar to that used for studying the systemic response and biodistribution experiments. In this model tumor cells are orthotopically implanted in the suprarenal area of wild type mice and therapy is given at the same time from tumor initiation.

**Figure 4:**
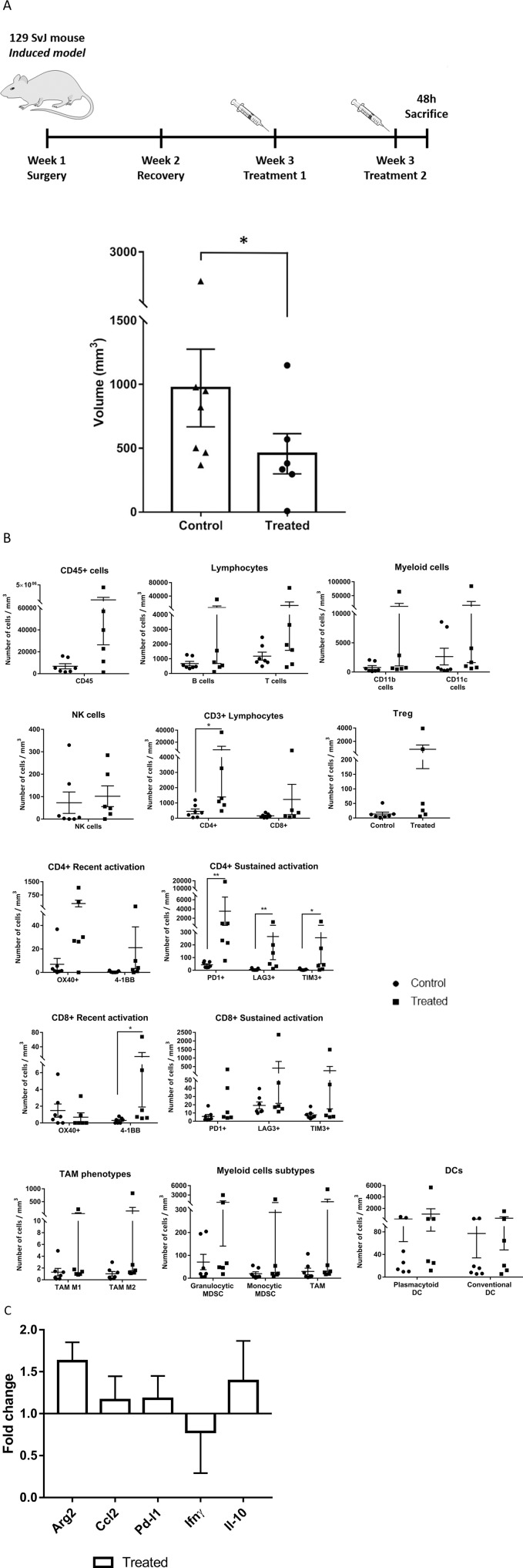
Effects of systemically administered mCelyvir in tumors in an induced model of NB. **(A)** Tumor volumes from TH-MYCN recovered after two weekly treatments of intravenous mCelyvir and untreated controls. **(B)** Infiltrating immune cells per milliliter of tumor in both groups of animals. **(C)** Expression levels of immune-related genes of tumor microenvironment analyzed by qRT-PCR. Results were normalized to untreated controls (normalized value = 1). Statistics differences are represented by ^*^ (p < 0.05) or ^**^ (p < 0.01).

Mice were treated and processed as explained before. All the animals showed macroscopic tumors in the area where cells were implanted. We found that tumors from treated mice (n=6) were significantly smaller than those of untreated controls (n=7) (Figure 4A, p=0.045, and Supplementary Figure 5). We also analyzed intratumoral immune populations by flow cytometry. We normalized the number of immune cells per milliliter of tumor to allow for comparisons. Tumors from treated mice had significantly higher immune infiltration compared to untreated ones, and this difference affected to all major leukocyte subpopulations (Figure 4B). Systemic therapy with mCelyvir resulted in increased numbers of CD4 T lymphocytes, expressing markers of recent (OX40, 4-1BB) and sustained T cell activation (PD1, LAG3 and TIM3) compared to untreated mice. The same was found for CD8, except for the expression of OX40, which was higher among control mice. CD4 and CD8 TILs expressing 4-1BB were enriched 9 and 55-fold respectively, relative to untreated mice (Supplementary Table 2). Subsets of MDSCs (granulocytic and monocytic), TAMs (TAM1 and TAM2) and DCs (plasmacytoid and conventional) were also higher in treated mice compared to control ones.

Finally, we performed qRT-PCR analysis to determine changes in expression of TME genes. The main changes found in gene expression levels affected *Arg2* (higher levels among treated mice) (Figure 4C).

## DISCUSSION

Oncolytic virotherapy can be considered as a form of cancer immunotherapy [4], since the reported clinical benefits have been associated with antitumor immune phenomena initiated by infection and oncolysis. The role of the immune system during treatment with oncolytic viruses has two different facets, with a direct impact on the results of the therapy. On the one hand, antiviral immunity may be an important potential limitation. It has been described that natural killer (NK) cells interfere with the action of oncolytic viruses, decreasing and even eliminating their efficacy [17]. It is also known that adaptive immunity has developed many mechanisms to destroy viral infections [18]. On the other hand, oncolysis causes the death of tumor cells with the possible release of tumor-associated antigens. These antigens, together with the danger signals [19] associated with viral infection, can stimulate an antitumor immune response [20] that increases the clinical effect in a very significant way.

The fact that MSCs are used for the production of Celyvir has very important implications in the development of immune responses that occur in these patients, which makes them different from those uses of virotherapy without MSCs. The effects that MSCs have on antigen-presenting cells (APCs, including DCs) and immune effector cells are well known [7–14]. It has also been reported that MSCs in the autologous context and in animal models can function as APCs [21, 22]. Therefore, the MSCs of Celyvir could exert an immunomodulatory role, participate in the presentation of antigens, or in both processes. In principle, MSCs infected with adenoviral vectors have not shown great changes in their phenotypic or functional characteristics [23]. From the point of view of the clinical practice with Celyvir, the MSCs could diminish the antiadenoviral response, due to its immunomodulatory role, facilitating and thus increasing the oncolytic action of the virus. In addition, the use of repeated administrations of an adenovirus (the regimen of treatment with Celyvir consists of weekly doses) could contribute to the depletion of the antiadenoviral immune response, comparable to that described in patients with other viral infections [24].

We present here a model that allows the study of many important aspects related to the Celyvir strategy we are developing in children with cancer. The TH-MYCN mouse recapitulates the main genetic and clinical characteristics of NB with amplified MYCN, and has been used over the last 20 years in many studies of basic biology and preclinical research [25]. One aspect that has not been intensively studied in this model is the profile of infiltrating immune cells of the tumors, both at baseline and after the administration of treatments. This point is very important in the case of immunotherapies. In the TH-MYCN mouse tumors develop in an immunocompetent host, with the appropriate tissue microenvironment, reproducing the conditions that allow the interaction of the immune system with the developing tumor.

We have treated these mice with a therapeutic regime similar to that we use in patients, i.e., an oncolytic adenovirus carried in MSCs, administered repeatedly through systemic infusions. MAV-1 is a murine oncolytic adenovirus comparable to ICOVIR-5 [1]: MAV-1 and ICOVIR-5 have genetic modifications that allow them to replicate preferentially in cells in which retinoblastoma (Rb) pathway is deregulated [15], a common feature of advanced human cancers (including neuroblastoma, NB) but not in healthy cells; MAV-1 and ICOVIR-5 are also able to replicate in MSCs, although at significant lower levels compared to replication in tumor cell lines [1] (Supplementary Figure 1A, 1B, 1C). We confirm an excellent safety profile for this therapy in mice, with no adverse effect or discomfort in the recipients (as happens in human patients [1–3]). Compared to naked virus, the repeated administration of mCelyvir resulted in relative accumulation of the oncolytic virus in organs such as spleen and lung, likely due to the sifting effect of these organs for the infused cells. Interestingly, mCelyvir also resulted in higher amount of virus in the tumor masses compared to the infusion of naked viral particles, underscoring that the carrier cells outcompeted the direct administration in oncolytic virotherapy. These results would indicate that Celyvir could efficiently target tumors metastasizing into the lungs, such as pediatric sarcomas or some adult carcinomas. The protection that MSCs could provide to the oncolytic adenovirus did not preclude the development of an antiadenoviral immune response, as manifested by our results not only in murine models but also in our experience in human patients [2]. However, the presence of circulating antiadenoviral antibodies did not prevent the changes detected at the tumor masses.

Less than half of the TH-MYCN mice developed a tumor during the time we did the experimental work, as it has been previously reported [16]. In addition, the time of tumor initiation is not homogeneous in this spontaneous model. Most mice develop tumors between weeks 6 and 9 of life, predominantly in the paraspinal region of these mice, and can be palpated only when they are large enough, which makes very difficult to know when a tumor is initiating on these mice. Also, no image system was available to follow up the development of these tumors and therefore choose the best moment to start treatment. All these facts pose limitations and account for a high variability of results when therapies are administered at the same starting time-point. Therefore, in addition to the spontaneous model we used an induced one, in which tumor cells are orthotopically implanted in the suprarenal area of 129/SvJ wild type mice [26–28]. In the latter, conditions such as frequency of tumors and time of tumor initiation are similar among animals in treated and control groups. Using any of the models we found that the systemic administration of mCelyvir resulted in local changes in the tumor masses related to their immune landscape. Since we did not find major changes in circulating leukocytes (Supplementary Figure 4), the scenario at the tumor represents a localized rather than generalized effect. Therapy caused an increased infiltration of immune cells per unit of tumor volume when compared to untreated mice. The recruitment of immune cells has been already reported when oncolytic viruses were administered directly into the tumor masses [29, 30] and our results show that the same happens when carrier cells systemically deliver the virus. We could also prove the presence of oncolytic virus in the tumor masses upon sacrifice.

Therapy also caused changes in the quality of immune cell infiltrates. Increased B and T lymphoid infiltration was independent of the type of model analyzed, while myeloid and DC subsets were affected mainly in the induced model. Others have reported that intratumoral administrations of oncolytic viruses in mice [29, 31] induced inflammatory responses with activation of CD4 and CD8 T lymphocyte infiltration in treated lesions. We also found that infiltrating CD4 and CD8 lymphocytes showed changes in markers of T cell activation and exhaustion upon repeated mCelyvir administration [32], suggesting continued activity of the tumor infiltrating T lymphocytes (TILs). TILs expressing activation markers were more enriched (relative to untreated mice) in the induced model, both CD4 and CD8, while the spontaneous model showed enrichment of CD4 and CD8 expressing exhaustion markers. Although we did not test the specificity of TILs activity in this work (antitumoral / antiadenoviral / non-specific), the immunophenotypic differences suggest a more active and less exhausted TIL repertoire in the induced model after mCelyvir therapy, which might explain the better antitumoral effect seen in terms of tumor volume.

We also analyzed changes related to the tumor microenvironment, specifically of molecules with a known role in the balance pro and antitumoral that modulates tumor growth. Systemic administration of mCelyvir was followed by changes in the TME, more remarkable in the spontaneous model than the induced one. After treatment, TME showed a less pro-tumoral and more inflammatory profile, a scenario that contributes to create a more pro-immune local situation for an antitumor immune response, as has been previously described [33, 34]. Today it is well accepted the immunomodulatory role associated to oncolytic virotherapy through several secondary mechanisms derived from the tumor cell infection itself [6]. In our experience, TME changed towards a more inflamed environment, evidenced by the increase of cytokines like CXCL10 and CCL2 [35, 36], and the decrease of immunosuppressive molecules (like FoxP3 and Nos2), and molecules that promote tumor growth and invasiveness (like IL10 and TGFβ) [37–39]. Therefore, oncolytic virotherapy with Celyvir could have not only a lytic effect in tumor cells, but could also immunomodulate the TME so other therapies can be administered with a higher chance of achieving a clinical response.

We are using the Celyvir strategy to treat kids with metastatic tumors [1–3]. We have found changes in the immune landscape of primary tumors of patients receiving Celyvir, similar to those described in this paper [27]. We found differences in immune cell infiltration and gene expression levels when comparing the spontaneous versus induced model, considering only non-treated mice. There were higher levels of immune infiltration and levels of gene expression (pro- and anti-tumoral genes) in the non-treated mice of the spontaneous model (Supplementary figure 6A, 6B). These differences may likely be due to the extended time and more physiological process for tumor and TME development in the spontaneous model, and the higher numbers of tumor initiating cells in the induced one. The impact of mCelyvir therapy in each scenario was different, changes in infiltration and TME more pronounced in the spontaneous model and tumor volume in the induced one. These differences in the response to mCelyvir may be related to the different doses used in each model. Nevertheless, even though these two models are not completely identical, and taking into account differences in the murine and human oncolytic adenoviruses, we believe that the models described here will help us in optimizing this type of therapy by enabling the study of crucial events related to the immune responses affecting both the medicine and the tumor.

## MATERIALS AND METHODS

### Mice

TH-MYCN- 129X1/SvJ transgenic mice [40] were used for spontaneous tumor treatment experiments. Wild type mice were also used for mouse NB tumor cell line transplant experiments. All mice were bred, maintained and used following guidelines issued by the European and Spanish legislations for laboratory animal care. All experiments involving animals were approved by the OEBA (Organ for Evaluating Animal Wellbeing) at CIEMAT and Madrid Regional Department of Environment, with reference PROEX 186/15. Transgenic mice were identified as previously described [41] using N008 (5'-TGGAAAGCTTCTTATTGGTAGAAACAA-3') and N009 (5'-AGGGATCCTTTCCGCCCCGTTCGTTTTAA-3') for human MYCN gene detection.

### Cell culture and virus production

WT 129/SvJ mice derived mesenchymal stem cells (mMSC) were obtained from adipose tissue enzymatically digested with 1 mg/mL of collagenase B (Roche; Catalog #11088815001) during 2 hours at 37ºC in constant shaking. Mononuclear cells were washed twice with PBS and pelleted by centrifugation at 1500 rpm for 5 minutes. Cells were seeded at 100.00 cells / cm^2^ with murine MesenCult Expansion Kit (STEMCELL Technologies; Catalog #05513) until passage 3. From then on mMSC were maintained with DMEM with Glutamax (Gibco) supplemented with 10% FBS (HyClone, GE Healthcare Life Sciences) and 1% penicillin / streptomycin (10.000 U/mL, Gibco; Catalog #15140122). Murine MSC were characterized by flow cytometry with specific antibodies.

Non-adherent spheres derived from a TH-MYCN tumor mass (36769 cell line) were generated in Dr. Louis Chesler’s laboratory. They were maintained using DMEM/F12 (Gibco; Catalog #10565018) supplemented with 1X B-27 without vitamin A (Gibco; Catalog #12587010), 20 ng/mL of murine EGF and 40 ng/mL of murine FGF (R&D Systems, Minneapolis, MN). Spheres were passed when confluents, and seeded again at 1/6 of its initial concentration.

The 37.1 cell line was used for MAV-1 dlE102 virus production. The oncolytic murine adenovirus MAV-1 dlE102 (mAd) was previously developed by Dr. Katherine Spindler’s group [15]. Similar to ICOVIR-5 [1], MAV-1 has genetic modifications that allows it to replicate preferably in cells in which retinoblastoma (Rb) pathway is deregulated [15]. First virus aliquot was kindly provided by Dr. Spindler. Successive virus stocks for our experiments were produced in our lab using 37.1 cells as previously described by Spindler group. The cells were maintained with DMEM (4.5 g/L glucose, Gibco; Catalog #11965092) supplemented containing 5% FBS (Hyclone, GE Healthcare Life Sciences) and G418 (200μg/mL, Sigma; Catalog #G8168-10ML). For virus propagation, cells were passaged one time in absence of G418, and 10^-5^M dexamethasone was added 5-24 hours prior infection to induce E1A expression [15]. Viral physical titer from supernatants was determined by spectrophotometry using NanoDrop 1000 (Thermo Fisher Scientific, Waltham, MA USA) and aliquoted and stored at -80ºC until use.

### Preparation of murine Celyvir

For murine Celyvir (mCelyvir) preparation, mMSC were used between passages 5 and 7 for every experiment. Cells were trypsinized (TripLE Express, Life Technologies; Catalog #12604013) and counted with trypan blue. Viable mMSC were infected at MOI 200 with MAV-1 dlE102 during 90 minutes at 37º C in constant shaking. After that, cells were washed to remove virus excess and resuspended for intravenously (IV) administration in mice (100.000 infected cells in 150 μL of non-supplemented DMEM).

### Tumor mice model and mCelyvir treatment

We either used the TH-MYCN transgenic mouse model (spontaneous) or implanted 3 x 10^5^ TH-MYCN cells on the suprarenal region of 129/SvJ mice (genetic background of the TH-MYCN transgenic mouse, induced model).

All treatments were administered intravenously, either mCelyvir or naked adenovirus.

In the spontaneous model, 8 weeks old TH-MYCN mice were treated intravenously with mCelyvir during 4 weeks while control group remained untreated. Both groups of mice were sacrificed two days after the 4^th^ treatment and peripheral blood and tumor mass were obtained.

For the induced tumor model, 3 x 10^5^ cells from a single cell suspension of the 36769 cell line were inoculated into the left adrenal gland of 129/SvJ-WT mice by surgery. Mice were left to recover for 2 weeks and then received 2 weekly doses of mCelyvir or remained untreated. Mice were sacrificed two days after the second treatment and peripheral blood and tumor mass were analyzed.

Tumor volume was calculated with the formula (3.14/3)×(a/2)^2^×(b/2). A tumor piece was conserved at -80ºC for qRT-PCR analysis. The rest of the tumoral mass and the peripheral blood were processed freshly for flow cytometry analysis.

### Biodistribution and viral replication studies

Mice with implanted tumors were treated intravenously during 3 weeks with mCelyvir or naked adenovirus once per week. The amount of naked mAd used was equivalent to that used in Celyvir preparation. After the last treatment, mice were sacrificed and bone marrow, spleen, lung, liver, kidney, tumor mass and peripheral blood (PB) were extracted to further analyze the presence of viral DNA in the different tissues.

For detection of mAdv presence on tumors, conventional PCR reaction was performed with 30 ng of genomic DNA using GoTaq® DNA Polymerase (Promega, Madison USA; Catalog #M7822) and specific MAV-1 forward (5’-GGCCAACACTACCGACACTT-3’) and MAV-1 reverse (5’-TTTTGTCCTGTGGCATTTGA-3’) primers. Conventional PCR reaction consisted in an initial denaturation at 95ºC for 10 minutes followed by 40 cycles at 95ºC for 15 seconds and 65ºC during 1 minute, as previously described [42].

To study the kinetics of viral particles in peripheral blood, treated mice were bleed just before each treatment and also one-two days after. qRT-PCR was performed using hexon (MAV-1) and ApoB (as genomic control) primers. The same qRT-PCR procedure was used to quantify adenovirus in distinct organs (bone marrow, spleen, lung, liver, kidney). The reactions were run in a 7500 Real-Time PCR System (Applied Biosystems™, California USA) and results were analyzed using 7500 Real-Time PCR software (Applied Biosystems™, California USA). Standard curves of genomic DNA and MAV-1 DNA were used for quantitation purposes.

### Flow cytometry analysis

To confirm the phenotype of mMSC cells were stained with specific antibodies during 30 min at 4 degrees. MSC were positive for CD29, CD44, and Sca-1 and negative for CD45, CD11b, and CD14 (data not shown).

To analyze immune infiltrated cells, tumor mass was carefully washed with HBSS 1X and mechanically processed prior enzymatic digestion with 1 mg/mL of collagenase D (Roche; Catalog #11088858001). Tumor cell suspension was counted by trypan blue and 5 x 10^5^ alive cells were labelled with specific antibodies during 30 min at 4 degrees. Red blood cells were lysed using QuickLysis (Cytognos; Catalog #CYT-QL-1) for 20 min in darkness. Used antibodies are indicated in supplementary information (Supplementary Table 1). Samples were acquired in a FacsCanto II (BD, San Jose, CA) cytometer and analyzed using FacsDiva software.

### RNA extraction and qRT-PCR analysis

To analyze gene expression of different stroma genes, chemokines and immune-related molecules, 30 μg of each tumor piece conserved at -80ºC were mechanically disrupted and RNA extraction was performed with RNeasy Mini kit (Qiagen; Catalog #74106). RNA concentration was determined by NanoDrop 1000. For cDNA synthesis, 1 μg of RNA was retrotranscribed using SuperScript™ VILO™ cDNA Synthesis Kit (Invitrogen, Catalog #11754050). For qRT-PCR analysis, specific murine TaqMan assays (Applied Biosystems™, California USA) were used: *Gapdh* (Mm99999915_g1), *Arg1* (Mm00475988_m1), *Arg2* (Mm00477592_m1), *Ccl2* (Mm00441242_m1), *Cd80* (Mm00711660_m1), *Cd86* (Mm00444543_m1), *Cxcl10* (Mm00445235_m1), *FoxP3* (Mm00475162_m1), *Ifnγ* (Mm01168134_m1), *Il-10* (Mm00439614_m1), *Pdl1* (Mm00452054_m1), *Tgfβ* (Mm01178820_m1), *Vegf* (Mm01281449_m1) and *Nos2* (Mm00440502_m1).

qRT-PCR reaction was performed in a 7500 Real-Time PCR System (Applied Biosystems™, California USA) and results were analyzed using 7500 Real-Time PCR software (Applied Biosystems™, California USA). A relative quantification was used (DDCt method) using non-treated groups of mice as reference.

### Serological studies

Fresh blood collected from spontaneous model TH-MYCN mice at the moment of sacrifice was tested for detection of specific MAV-1 antibodies in serum using Mouse Adenovirus (FL/K87) ELISA Kit (Dynamimed, Madrid Spain). Experiment was performed following manufacturer’s instructions. Briefly, peripheral blood samples were collected from animals and serum was obtained by centrifugation and diluted 50 times in order to proceed with ELISA. Samples were incubated in the plate as well as proper positive and negative controls at 37ºC during 45 minutes. Wells were carefully washed five times before adding peroxidase conjugate and incubate again at 37ºC during 45 minutes. After last five washes, peroxidase substrate was added and plate was incubated for 30 minutes prior to read the absorbance at 495 nm.

### Statistics

All statistical analyses were performed using Stata/IC 11.0 (StataCorp LP, College Station, TX, http://www.stata.com/). The nonparametric Wilcoxon rank-sum test was used to compare quantitative variables. Results were considered statistically significant with p<0.05. All graphics present the mean ± SEM.

### Graphics

All graphics presented in this work have been created using GraphPad Prism 7 (https://www.graphpad.com/).

## SUPPLEMENTARY MATERIALS FIGURES AND TABLES



## Abbreviations

mCelyvir: murine Celyvir
IV: intravenous / intravenously
TME: tumor microenvironment
NB: neuroblastoma
MSC: mesenchymal stem cells
qPCR: quantitative polymerase chain reaction.
