# Supplementary material for: Systemic oncolytic adenovirus delivered in mesenchymal carrier cells modulate tumor infiltrating immune cells and tumor microenvironment in mice with neuroblastoma
Source: Oncotarget. 2020 Jan 28;11(4):347–61. doi: 10.18632/oncotarget.27401 (PMC6996901; doi:10.18632/oncotarget.27401)
Supplement: Supplementary file 1 [file oncotarget-11-347-s001.pdf]

Systemic oncolytic adenovirus delivered in mesenchymal carrier cells modulate tumor infiltrating immune cells and tumor microenvironment in mice with neuroblastoma

SUPPLEMENTARY MATERIALS

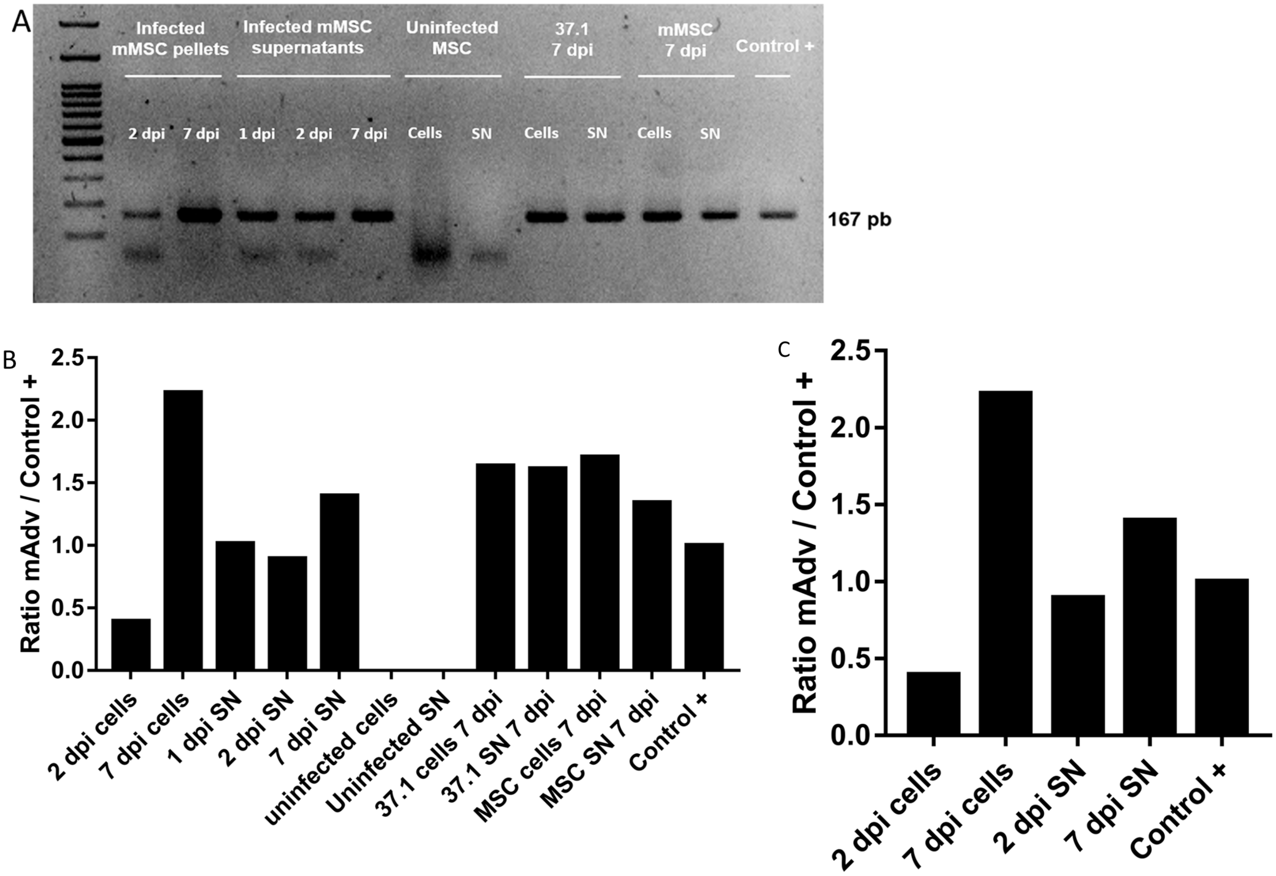

Supplementary Figure 1: Mutant oncolytic adenovirus dlE102 replicates efficiently in adipose-derived murine MSC. (A) Conventional PCR was performed from genomic DNA using MAV-1 primers**.** Infected mMSC pelleted cells and its supernatants (SN) were evaluated two and seven days after infection. Infected 37.1 cells and supernatants are shown for comparisons. Positive control sample comes from a successful viral production. **(B)** Graphical representation of a semi-quantitative approach of the PCR showed in A. Quantification was made using ImageJ software and positive control was used to calculate ratio for each sample. Data was normalized to control, so reference value is 1 (this value corresponds to positive control). **(C)** Graphical representation showing replication rates only for mMSC at distinct times. All experiments were performed once.

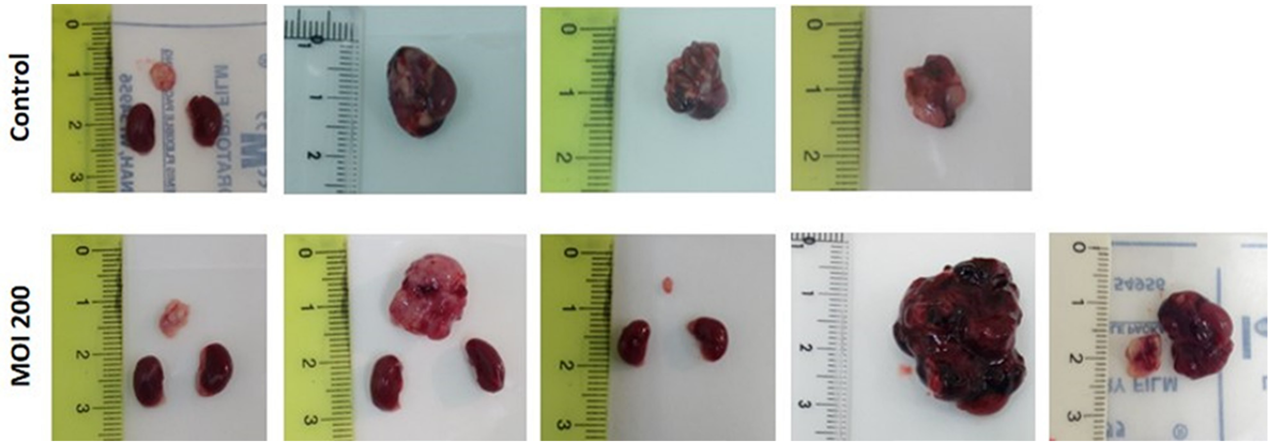

Supplementary Figure 2: Spontaneous TH-MYCN tumors.

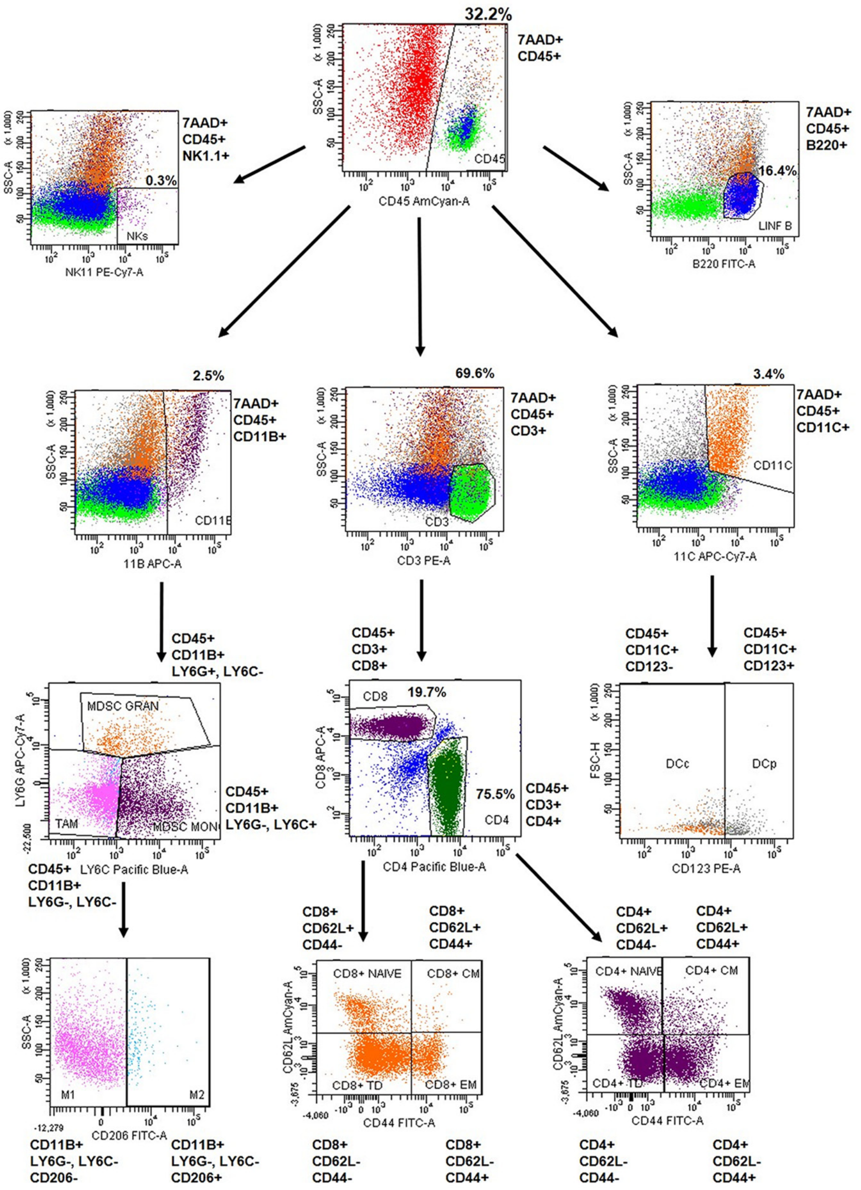

Supplementary Figure 3: Flow cytometry strategy scheme for tumor infiltrating immune cell analysis. (EM: Effector memory; TD: Terminally differentiated; CM: Central memory; NK: Natural killer; DC: Dendritic cells; TAM: Tumor associated macrophages; MDSC: Myeloid derived suppressor cells).

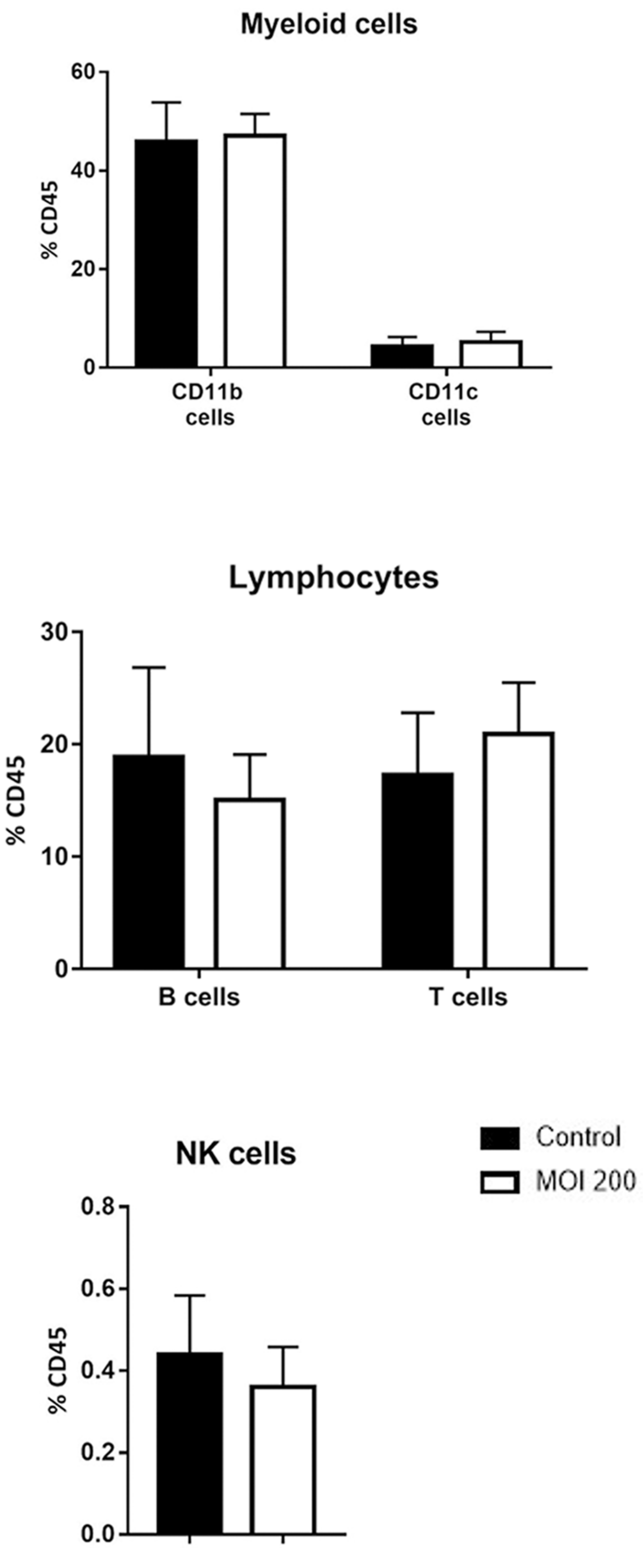

Supplementary Figure 4: Total percentage of leukocytes in peripheral blood (PB) of TH-MYCN mice from spontaneous tumor model.

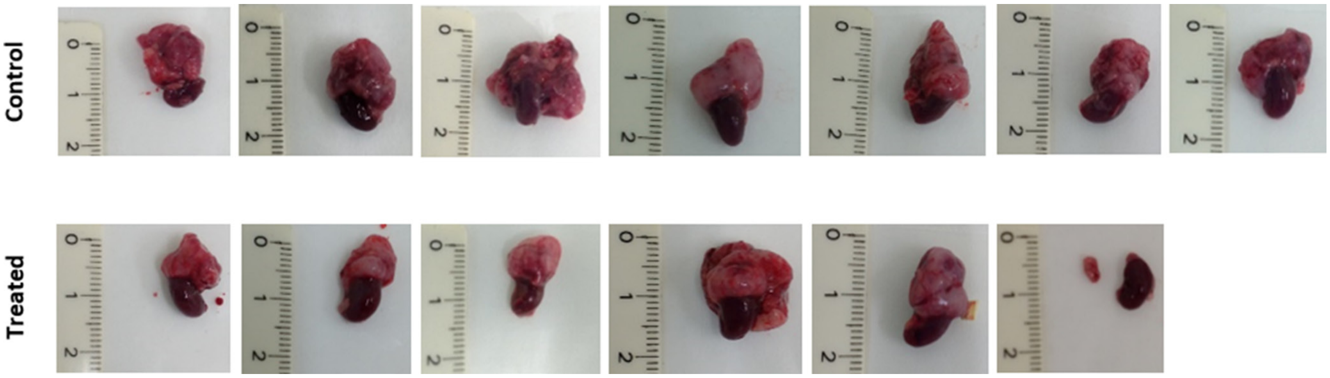

Supplementary Figure 5: Induced TH-MYCN tumors.

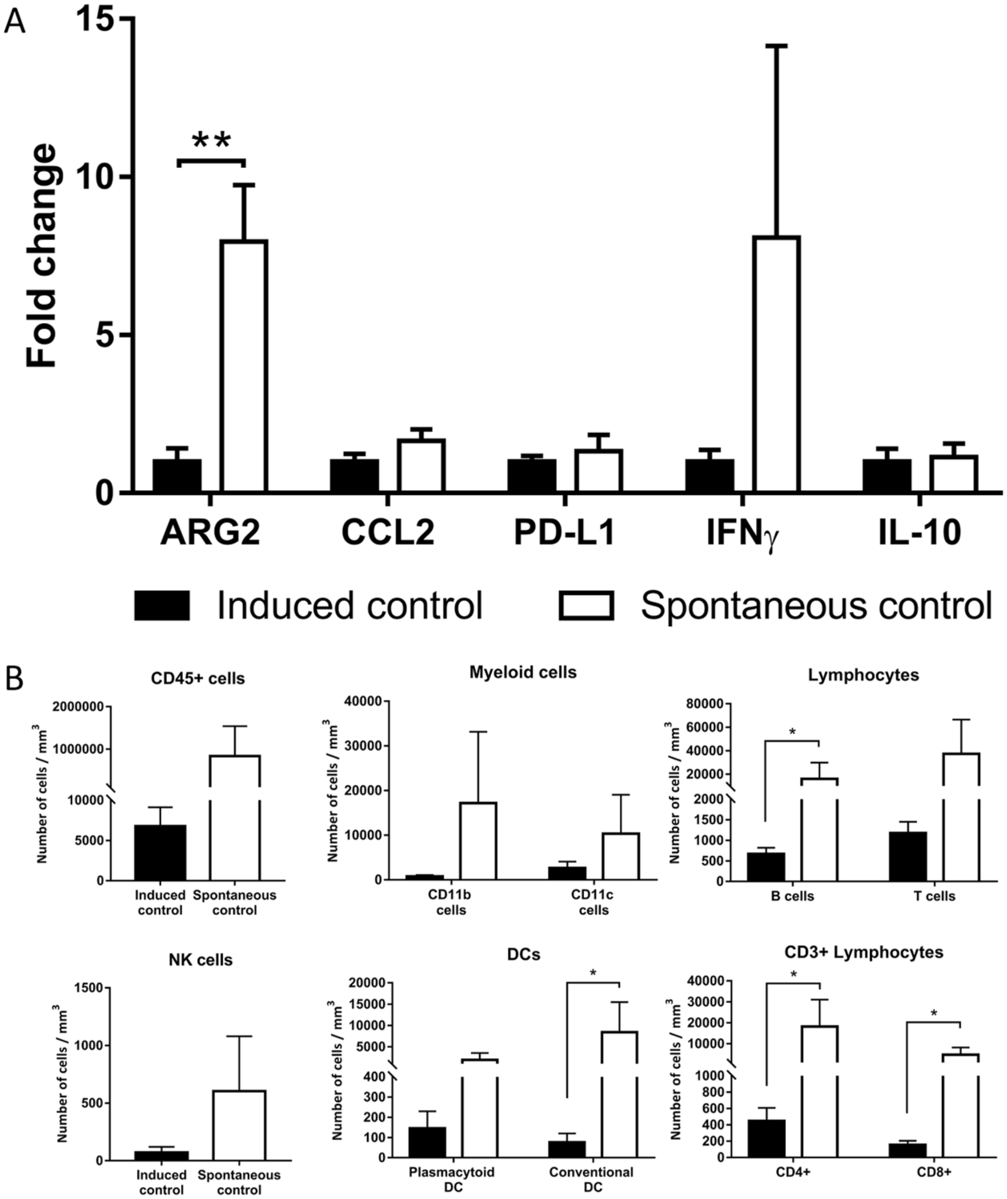

Supplementary Figure 6: Comparation between control (untreated) mice from the induced and the spontaneous model of NB. (A) qRT-PCR comparison for common immune-related genes of the TME (ARG2, $p<0.01$). **(B)** Comparison between immune populations by flow cytometry. B cells, Conventional DCs, CD4+ cells and CD8+ cells show statistical differences between both groups ($p<0.05$).

Supplementary Table 1: Detailed list of antibodies used for flow cytometry studies

Antibody	Fluorophore	Reference	Company
**CD11b Antibody**	APC	101212	Biolegend
**CD8a Antibody**	APC	100712	Biolegend
**CD25 Antibody**	APC/Cy7	102026	Biolegend
**CD279 (PD-1) Antibody**	APC/Cy7	135223	Biolegend
**CD11c Antibody**	APC/Cy7	117324	Biolegend
**Ly-6A/E (Sca-1) Antibody**	APC/Cy7	108125	Biolegend
**Ly-6G Antibody**	APC/Cy7	127624	Biolegend
**CD14 Antibody**	APC/Cy7	123318	Biolegend
**CD45 Antibody**	Brilliant Violet 510™	103138	Biolegend
**CD62L Antibody**	Brilliant Violet 510™	104441	Biolegend
**CD103 Antibody**	FITC	121419	Biolegend
**CD206 (MMR) Antibody**	FITC	141704	Biolegend
**CD44 Antibody**	FITC	103022	Biolegend
**CD45R/B220 Antibody**	FITC	103206	Biolegend
**CD134 / OX40 Antibody**	FITC	MA5-17917	Fisher
**CD137 (4-1BB) Antibody**	FITC	558975	BD
**CD29 Antibody**	FITC	102206	Biolegend
**CD4 Antibody**	Pacific Blue™	100531	Biolegend
**Ly-6C Antibody**	Pacific Blue™	128014	Biolegend
**Ly-6A/E (Sca-1) Antibody**	Pacific Blue™	122520	Biolegend
**CD11c Antibody**	PE	117308	Biolegend
**CD122 (IL-2Rβ) Antibody**	PE	123209	Biolegend
**CD123 Antibody**	PE	106005	Biolegend
**CD223 (LAG-3) Antibody**	PE	125207	Biolegend
**CD3ε Antibody**	PE	100308	Biolegend
**CD11c Antibody**	PE	117308	Biolegend
**CD44 Antibody**	PE	103024	Biolegend
**CD152 (CTLA-4) Antibody**	PE/Cy7	106313	Biolegend
**CD366 (Tim-3) Antibody**	PE/Cy7	119715	Biolegend
**F4/80 Antibody**	PE/Cy7	123114	Biolegend
**CD95 Antibody**	Pe/Cy7	557653	BD
**NK-1.1 Antibody**	PE/Cy7	108714	Biolegend
**7AAD Viability Staining**	PERCP/CY7	420404	Biolegend

Supplementary Table 2: Ratios of each tumor infiltrating immune population (per tumor volume) for each mice model, normalized to corresponding controls

	Ratio between treated and non-treated mice in each murine model (Treated / Non-treated)	
	Spontaneous model	*Induced model*
CD45 / vol	2	5
LinfB / vol	5	5
LinfT / vol	3	6
CD11B / vol	0	8
CD11C / vol	1	3
NK / vol	1	7
CD4 / vol	8	3
Treg / vol	0	6
$CD4^+OX40^+$ / vol	1	4
$CD4^+41BB^+$ / vol	1	9
$CD4^+PD1^+$ / vol	150	7
$CD4^+LAG3^+$ / vol	7	1
$CD4^+TIM3^+$ / vol	10	5
CD8 / vol	6	7
$CD8^+OX40^+$ / vol	2	0
$CD8^+41BB^+$ / vol	0	55
$CD8^+PD1^+$ / vol	33	6
$CD8^+LAG3^+$ / vol	2	10
$CD8^+TIM3^+$ / vol	1	15
MDSC Gran / vol	0	4
MDSC Mono / vol	0	5
TAM / vol	0	8
TAM M2 / vol	0	19
TAM M1 / vol	0	5
DCp / vol	0	6
DCc / vol	1	3
